# Negative Regulation of TGFβ Signaling by Stem Cell Antigen-1 Protects against Ischemic Acute Kidney Injury

**DOI:** 10.1371/journal.pone.0129561

**Published:** 2015-06-08

**Authors:** Troy D. Camarata, Grant C. Weaver, Alexandr Vasilyev, M. Amin Arnaout

**Affiliations:** 1 Leukocyte Biology & Inflammation Program, Renal Division and Department of Medicine Massachusetts General Hospital, Charlestown, Massachusetts, United States of America; 2 Center For Regenerative Medicine, Department of Medicine, Massachusetts General Hospital, Charlestown, Massachusetts, United States of America; National Cancer Institute, UNITED STATES

## Abstract

Acute kidney injury, often caused by an ischemic insult, is associated with significant short-term morbidity and mortality, and increased risk of chronic kidney disease. The factors affecting the renal response to injury following ischemia and reperfusion remain to be clarified. We found that the Stem cell antigen-1 (Sca-1), commonly used as a stem cell marker, is heavily expressed in renal tubules of the adult mouse kidney. We evaluated its potential role in the kidney using Sca-1 knockout mice submitted to acute ischemia reperfusion injury (IRI), as well as cultured renal proximal tubular cells in which Sca-1 was stably silenced with shRNA. IRI induced more severe injury in Sca-1 null kidneys, as assessed by increased expression of Kim-1 and Ngal, rise in serum creatinine, abnormal pathology, and increased apoptosis of tubular epithelium, and persistent significant renal injury at day 7 post IRI, when recovery of renal function in control animals was nearly complete. Serum creatinine, Kim-1 and Ngal were slightly but significantly elevated even in uninjured Sca-1-/- kidneys. Sca-1 constitutively bound both TGFβ receptors I and II in cultured normal proximal tubular epithelial cells. Its genetic loss or silencing lead to constitutive TGFβ receptor—mediated activation of canonical Smad signaling even in the absence of ligand and to KIM-1 expression in the silenced cells. These studies demonstrate that by normally repressing TGFβ-mediated canonical Smad signaling, Sca-1 plays an important in renal epithelial cell homeostasis and in recovery of renal function following ischemic acute kidney injury.

## Introduction

Hospital-associated acute kidney injury (AKI) remains a significant clinical problem worldwide [1], affecting ~15% of all hospitalized patients [2, 3]. In the United States, more than 3 million hospitalized patients are at risk of AKI each year [4]. AKI once considered an incident from which patients generally recover, is now recognized as a major risk factor in progression of kidney disease, especially in predisposed individuals [5, 6].

Much of our understanding of the pathophysiology of AKI has been derived from animal studies of ischemia-reperfusion injury (IRI) induced by acute occlusion of the renal artery [7]. In rodents, IRI is associated with a rise in serum creatinine, induction of renal injury markers such as Kidney injury molecule-1 (Kim-1/Tim-1) and neutrophil gelatinase-associated lipocalin (Ngal)[8, 9] and epithelial cell death. These renal injury markers resolve in normal mice by day 7 following a single episode of acute IRI, but can persist in predisposed epithelium, resulting in interstitial fibrosis and chronic kidney disease [10]. The nature of the factors that affect the kidney response to acute IRI injury remain to be fully elucidated.

Stem cell antigen-1 (Sca-1, also called Ly6a), a member of the Ly-6 protein family [11]. is an 18-kDa glycerophosphatidylinositol (GPI)-anchored protein. Sca-1 is commonly used as a marker for the identification and isolation of stem cell and progenitor populations [12–16]. Sca-1 plays important roles in self-renewal and differentiation of stem and progenitor cells [11, 17, 18], in remodeling extracellular matrix during skeletal muscle regeneration [19], and in preservation of cardiac muscle function after pressure overload [20]. Sca-1 is also expressed in the adult kidney [21, 22], but its role there is unknown.

In this communication, we showed that Sca-1 is heavily expressed in renal proximal and distal nephron but not in the collecting ducts. We also elucidated its role and mechanism of action in normal renal tubular epithelium, using Sca-1 null and normal mice subjected to IRI and renal proximal tubular epithelium in which Sca-1 was stably silenced. We show that loss of Sca-1 in null mice lead to the expression of renal injury markers under baseline conditions, a more severe kidney injury and impaired renal recovery following renal IRI, compared to normal mice. Epithelial Sca-1 interacts with TGFβ receptors I and II (TβRI and TβRII, respectively) *in vitro* and *in vivo*, leading to the inhibition of TGFβ-induced canonical Smad signaling. These studies reveal a novel renoprotective role for Sca-1 in IRI, acting through suppression of TGFβ-directed canonical Smad signaling in renal tubular epithelium.

## Materials and Methods

### Animals

All procedures were approved by the Institutional Animal Care and Use Committee of Massachusetts General Hospital. Ly6a-EGFP (Sca-1GFP) mice were purchased from Jackson Laboratories (Bar Harbor, ME). Sca-1 knockout mice were provided by Dr. William Sanford (University of Ottawa) [23], and maintained on a C57BL6 background. C57BL6 wild-type animals were derived from a Sca-1 heterozygous knockout mating, and were bred in-house. Animals 10–12 weeks of age were used for all procedures. Severe ischemia/reperfusion injury (IRI) was performed on male mice using a non-traumatic microaneurysm clamp (Roboz surgical) placed on the left renal pedicle for 32 minutes while the right kidney was removed [24]. Animals were maintained at 37°C during surgery and recovery from anesthesia. Animals were sacrificed at the indicated time points, and the kidneys were perfused and harvested. Serum creatinine was analyzed using a mouse enzymatic creatinine assay kit (Crystal Chem).

### Cell culture

Immortalized mouse proximal tubule epithelial cells (TKPTS) were provided by Dr. Elsa Bello-Reuss (Texas Tech University), and cultured as described (Ernest and Bello-Reuss, 1995). Cells were maintained in DMEM/F12 media (Corning Cellgro) and supplemented with 7% FBS (Atlanta Biologicals) and 50 μU/ml insulin (Invitrogen). For Sca-1 silencing, TKPTS cells were infected with lentivirus containing shRNA-targeting Sca-1. shRNA clones D5 (GTGGGAGTAGTGTGTGAAATA) and C8 (AGGCAGCAGTTATTGTGGATT) displayed the most significant knockdown of Sca-1 (S2 Fig), and were used for further analysis. Sca-1 silenced cell lines were maintained in the above growth media and supplemented with 2μg/ml puromycin (Invitrogen). Cells were treated with recombinant human TGFβ_1_ (R&D Systems) at a concentration of 5ng/ml for the indicated times, and with the TβRI inhibitor SB431542 (Selleckchem) at 5μM concentration.

### Immunofluorescence and Imaging

Mouse kidneys were perfused and fixed in 4% formaldehyde prepared from paraformaldehyde and treated with 30% sucrose dissolved in PBS. Cryosections (7μm) were permeabilized with 1% Triton-X 100, washed in 0.5% BSA/PBS before blocking in 10% normal goat serum (NGS) diluted in 6% BSA/PBS. Tissue was incubated for 1 hour with primary antibodies diluted in 10% NGS, 6% BSA/PBS blocking buffer followed by 0.5% BSA/PBS washes. Secondary antibodies were diluted in 6% BSA/PBS and incubated for 1 hour followed by 0.5% BSA/PBS washes, and mounted using ProLong Gold (Invitrogen). TKPTS cells were fixed with 4% formaldehyde prepared from paraformaldehyde and were permeabilized with 0.5% Triton-X 100. Cells were incubated in blocking buffer containing 3% BSA/PBS followed by primary and secondary antibodies diluted in 1% BSA/PBS. Primary antibodies used were directed against Sca-1 and Smad2/3 (BD Biosciences), AQP1, Ki67 (Abcam), CD3, F4/80, Kim-1/Tim-1 (eBioscience), TβRI and TβRII (Santa Cruz), and Tamm-Horsfall protein (provided by Dr. John Hoyer). Cells were incubated with cell stains rhodamine *dolichos biflorus* agglutinin (DBA, Vector Laboratories), Alexa 555 phalloidin, Alexa 555 wheat germ agglutinin, and secondary antibodies Alexa 488 or 555 (all from Invitrogen). Cell death was detected using In Situ Cell Death detection kit, TMR red (Roche). Images were obtained using a Zeiss LSM510 or Zeiss LSM Pascal confocal microscope.

### Quantification of kidney injury

Tissue injury was scored on a 1–6 scale by adding parameters for severity and extent of tubular injury, as previously published [25]. The scores were assigned as follows. Severity of tubular injury: 0, no injury; 1, mild injury with mild attenuation of the epithelium and a loss of brush border on PAS stain; 2, moderate injury with marked attenuation of the epithelium but without frank denudation of basement membrane; and 3, severe injury with denudation of basement membrane. Extent of tubular injury: 0, no injury; 1, small isolated foci of injured epithelium; 2, confluent areas of injured epithelium but without uniform confluent involvement of corticomedullary junction; and 3, diffuse injury involving the entire cortico-medullary junction. Intermediate scores were assigned when appropriate, e.g. an isolated tubule with very mild injury would get a score of 0.5 + 0.5 = 1. Whole sagittal sections across the middle section of the entire kidney were from 2 normal and 3 Sca-1^-/-^ animals 7 days post-IRI, as well as from uninjured control and Sca-1^-/-^ animals were scored by the pathologist (A.V.) in a blinded fashion. For quantification of leukocyte infiltration into IRI kidneys, 10 high-powered fields were analyzed across two different kidney sections from each animal.

### Immunoprecipitation and Western blot analysis

Whole mouse kidney or cell protein lysates were obtained as described [26]. Briefly, flash frozen, homogenized tissue or cultured TKPTS cells were incubated with lysis buffer (25mM Tris-HCl, 100mM NaF, 10mM EGTA, 5mM EDTA, 250mM NaCl, 1% NP-40, 50mM Na_4_P_2_O_7_•H_2_O, 0.5% sodium deoxycholate (DOC), and 10mM ATP) containing protease inhibitors (Sigma) on ice for 20 min followed by centrifugation. Supernatants were collected and used for immunoprecipitation or Western blot. For immunoprecipitation, antibody-conjugated Protein A/G sepharose beads (Pierce) were incubated overnight with cell lysates at 4°C. The beads/lysate mix was washed three times in lysis buffer and protein was eluted with SDS buffer, boiled, and analyzed by Western blot. For Sca-1 immunoprecipitations, cells were washed in PBS and crosslinked using 3,3´-Dithiobis [sulfosuccinimidyl-propionate](DTSSP) (Pierce) according to the manufacturer instructions. In addition to the anti-Sca-1, Smad2/3, TβRI, TβRII and Kim-1/Tim-1, primary antibodies directed against p-Smad3, p-p38 and p38 (Cell Signaling), and α-tubulin (Sigma), were used, then detected using HRP conjugated secondary antibodies and SuperSignal chemiluminescence (Pierce). Quantification of protein band intensities was performed using ImageJ [27] and compared between wild type and Sca-1 mutants.

### Real-time PCR

To isolate RNA from kidneys, tissue was minced and incubated with collagenase at 37°C for 30 minutes followed by RNA extraction using RNeasy plus kit (Qiagen). cDNA was produced using ProtoScript II reverse transcriptase (New England Biolabs). Real-time PCR was performed on Stratagene MX4000 using iQ SYBR Green supermix (Biorad).

### Statistical Analysis

The significance of the difference between experimental groups was determined by analysis of variance followed by a one-tailed Student’s t test. Data are expressed as the mean ± SD, with P-values of < 0.05 considered significant.

## Results

### Expression of Sca-1 in the kidney of adult mice

**Fig 1:**
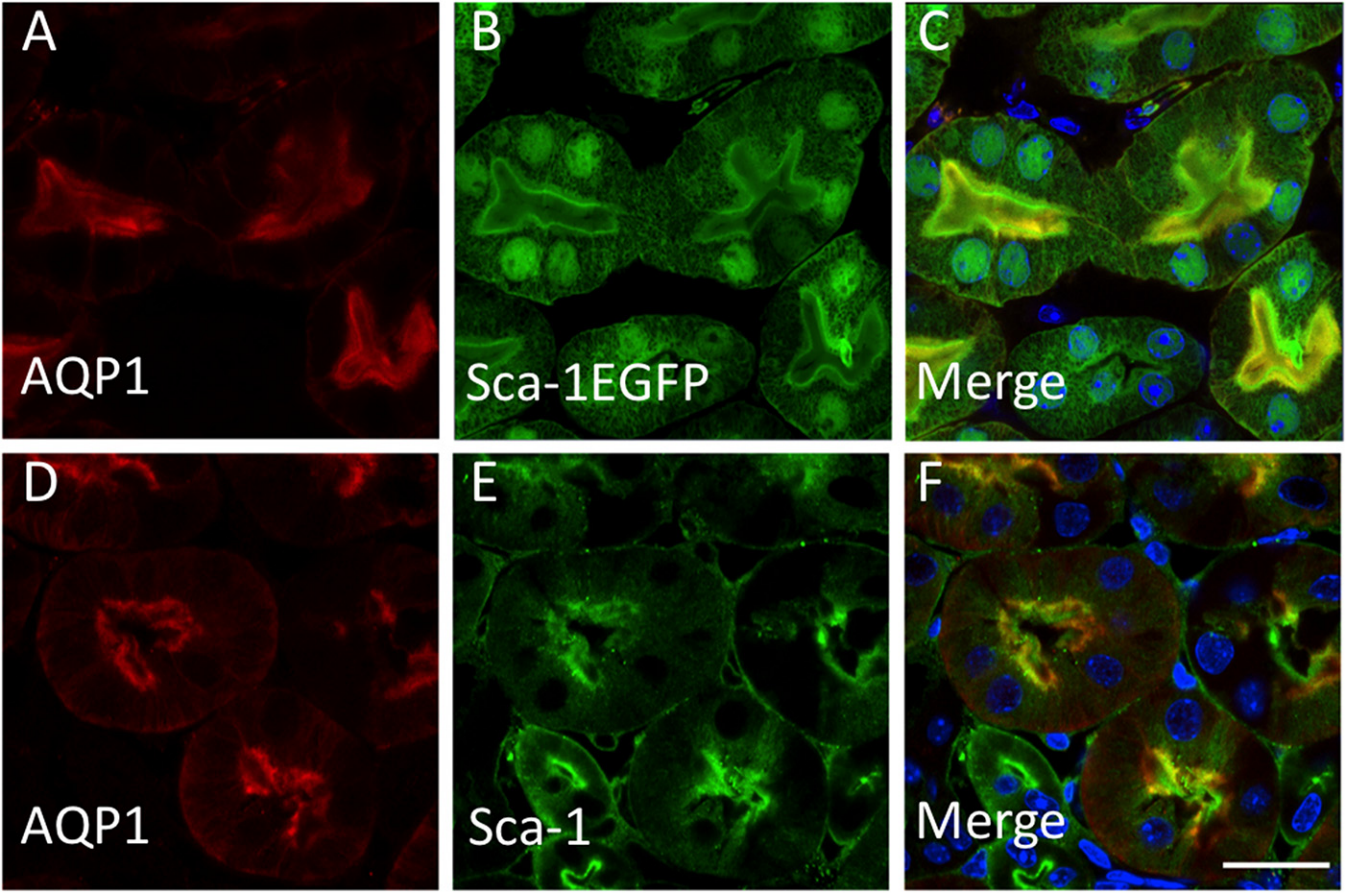
Sca-1 expression in adult renal epithelial cells. (A-C) Immunolocalization of proximal tubule marker AQP1 (A) in Sca-1-EGFP transgenic mouse (B). Merged image in C shows colocalization of AQP1 and Sca-1. (D-F) Immunolocalization of AQP1 (D) and Sca-1 (E) in normal adult mouse kidney using an antibody to each antigen. (F) Merged D and E images. Scale bar = 20μm.

Although Sca-1 (Ly6a) expression has been detected in the adult murine kidney sometime ago [21, 22], a detailed description of its tissue distribution has not been undertaken. To determine which renal cells express Sca-1, we utilized a transgenic mouse with EGFP under the control of the Sca-1 promoter, as well as Sca-1-specific antibody immunofluorescence. In Sca-1-EGFP transgenic mice, EGFP expression was prominently detected in tubular epithelium of proximal tubules (Fig 1A–1C), loop of Henle (S1A–S1C Fig), and distal tubules (S1D–S1F Fig), but not in the collecting duct (S1G–S1I Fig). Similar results were obtained using a Sca-1 antibody (Fig 1D–1F, and data not shown), which also revealed the apical localization of Sca-1 protein on proximal tubular cells (Fig 1D–1F).

### Loss of Sca-1 increased kidney injury following IRI

**Fig 2:**
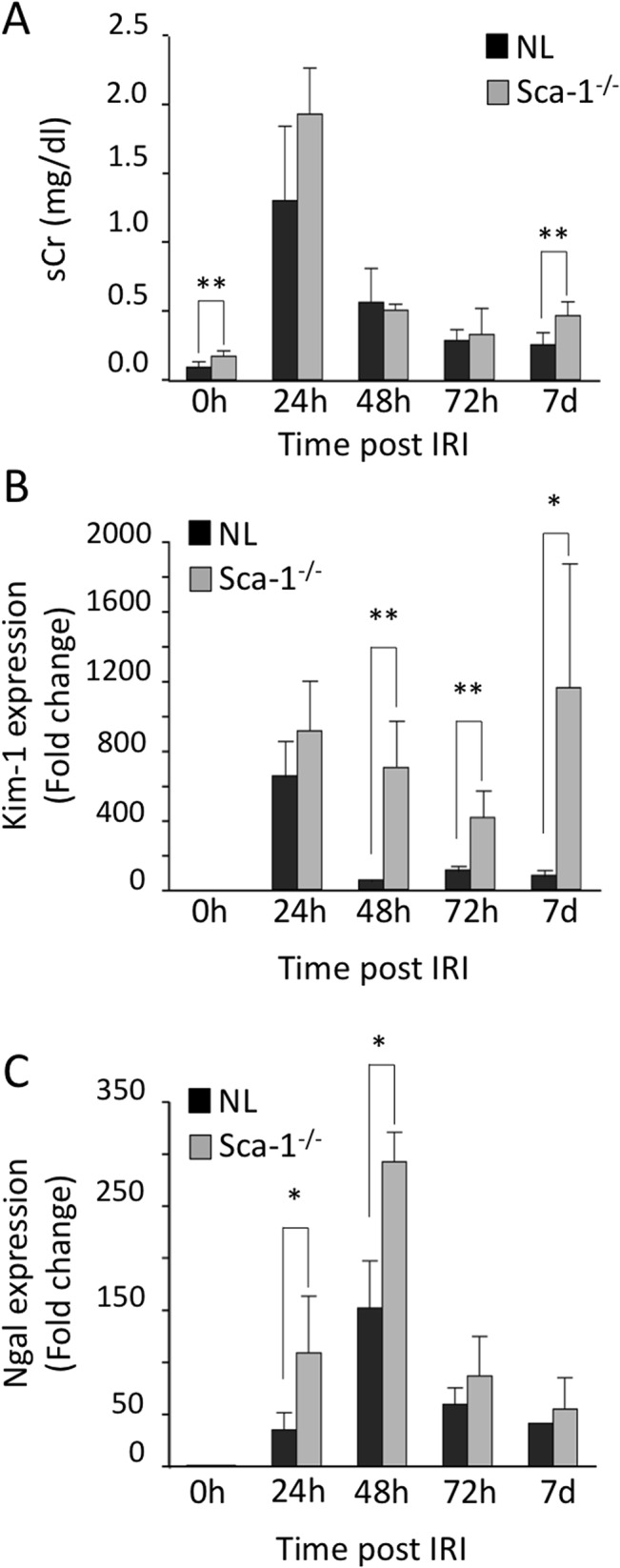
Changes in renal injury markers in normal (NL) and Sca1^-/-^ animals after IRI. (A) Serum creatinine measurements in normal (NL) and Sca1^**-/-**^ animals prior to (0h) and at different times following IRI. Significant differences were found between animals at 0h (uninjured)(p = 0.0019), and at day 7 post injury (p = 0.002). (B and C) Comparisons of fold induction of *Kim-1* and *Ngal* mRNA as measured by quantitative PCR (qPCR) in kidneys from Sca1^**-/-**^ vs. NL animals. Kim-1 (B): p = 0.0036 (48h), p = 0.0013(72h), p = 0.0071(7d); Ngal (C): p = 0.0128 (24h), p = 0.0056 (48h)). *, p<0.05; **, p<0.005; ***, p<0.0005. Normal control animals: n = 6 (0h), 6 (24h), 4 (48h), 5 (72h), 5 (7d). Sca1^**-/-**^: n = 6 (controls), 4 (24h), 3 (48h), 6 (72h), 7 (7d).

Kidney development appeared normal in Sca1^-/-^ mice, and kidneys from adult Sca1^-/-^ mice were visually similar to wild-type kidneys (data not shown), suggesting that Sca-1 plays little role in kidney development. To explore the potential role of Sca-1 in the adult kidney, we evaluated renal function following unilateral ischemia/reperfusion injury (IRI) with contralateral nephrectomy. Similar to wild-type animals, Sca-1^-/-^ animals displayed peak serum creatinine levels 24 hours following IRI, and began to recover toward baseline levels by 72 hours post-IRI (Fig 2A). However, serum creatinine levels rose again in Sca-1^-/-^ mice by day 7 post-IRI (0.47 mg/dl ± 0.1; Fig 2A), when wild-type mice were showing further recovery (0.26 mg/dl ±0.09, p = 0.002; Fig 2A). Interestingly, even uninjured Sca-1^-/-^ mice displayed a slight but significant elevation in levels of serum creatinine when compared to uninjured wild-type mice (0.170 ± 0.04 mg/dL and 0.093 ± 0.04 mg/dL, respectively; p = 0.0019)(Fig 2A). Sca-1 heterozygotes behaved similar to wild-type mice after IRI (unpublished data).

**Fig 3:**
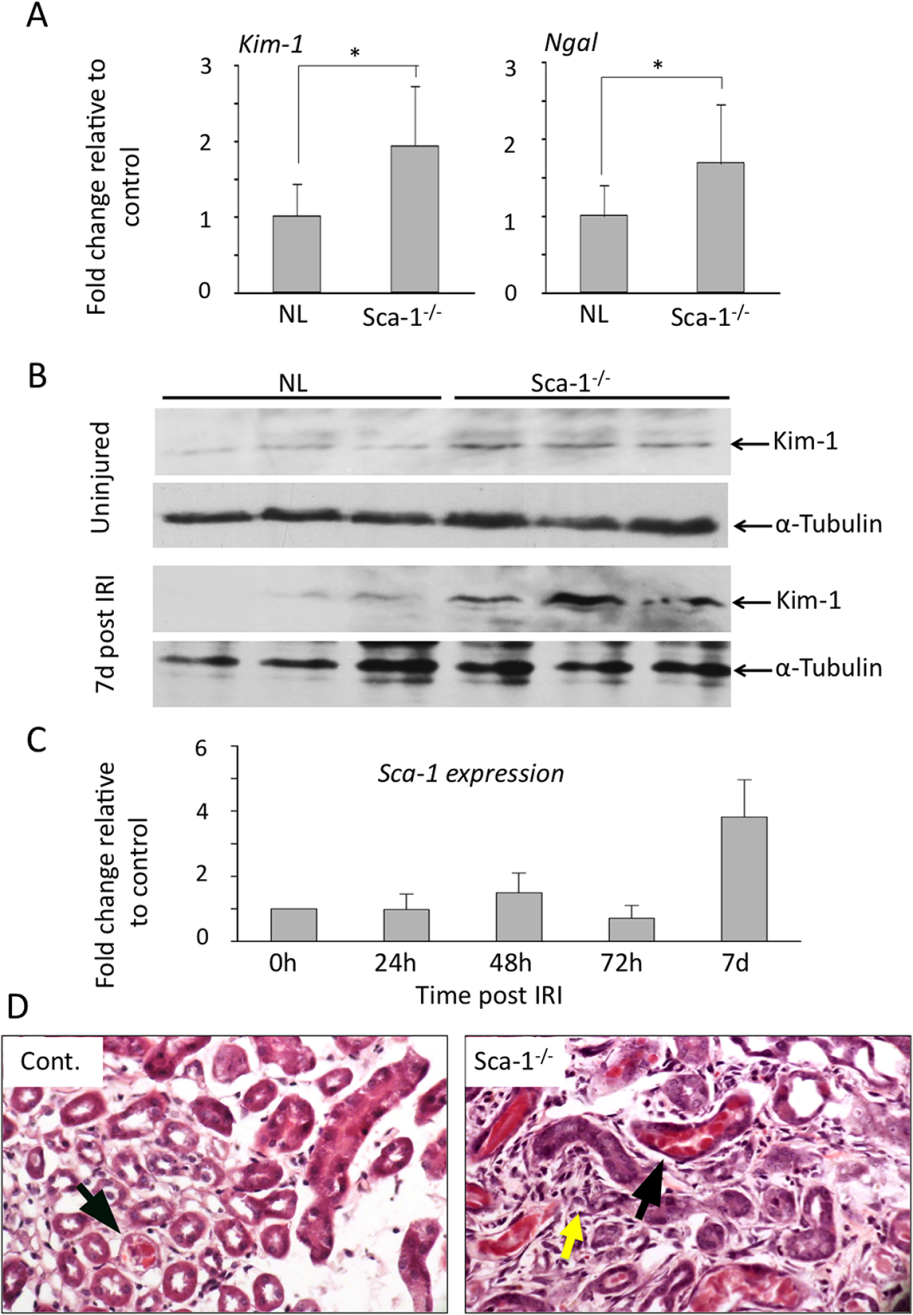
Sca1^-/-^ kidneys display increased injury after IRI. (A) Histograms (mean+sd) comparing *Kim-1* and *Ngal* mRNA levels in uninjured NL and Sca1^**-/-**^ kidneys. p = 0.0258 and 0.0227, respectively. (B) Western blot detection of Kim-1 protein in uninjured kidneys and in kidneys 7 days post-IRI from three NL and Sca1^**-/-**^ animals. Kim-1 was significantly induced kidneys in uninjured kidneys (upper panel) and in IRI kidneys at day 7 (lower panel) post IRI in Sca1^**-/-**^ mice vs. NL mice (p = 0.0183 and p = 0.010, respectively). (C) Histograms (mean+sd) showing qPCR measurement of *Sca-1* expression in wild-type kidneys after IRI. Normal control animals: n = 6 (0h), 6 (24h), 4 (48h), 5 (72h), 5 (7d). Sca1^**-/-**^: n = 6 (controls), 4 (24h), 3 (48h), 6 (72h), 7 (7d). (D) High power H&E stain of representative 6 μm sections from control (Cont.) and Sca1^**-/-**^ kidneys 7 days post IRI. Tubular casts (black arrows) and increased interstitial cellularity (yellow arrow) were frequently observed in Sca1^**-/-**^ kidneys. Kidney injury scores (mean+s.d.) 7 days post IRI were 3.0 and 2.75 for two controls and 4.5, 3.5 and 3.5 for three Sca1^**-/-**^ mice. Injury scores of zero were derived for kidneys from uninjured control and Sca1^**-/-**^ mice.

Both *Kim-1* and *Ngal* mRNAs were highly induced following injury in wild type and Sca-1^-/-^ kidneys (Fig 2B and 2C). However, whereas *Kim-1* and *Ngal* mRNA levels progressively declined in wild-type animals over the next 6 days, high levels of *Kim-1* expression persisted in Sca-1^-/-^ mice throughout the first week following injury. By day 7 post-IRI, Sca-1^-/-^ kidneys had a greater than 10-fold increase in *Kim-1* expression compared to wild-type kidneys (Fig 2B), indicating impaired renal recovery. Low but significant levels of *Kim-1* and *Ngal* mRNA were detected in uninjured Sca-1^-/-^ kidneys (Fig 3A), consistent with elevated serum creatinine (Fig 2A), suggesting that Sca-1^-/-^ kidneys are intrinsically susceptible to IRI. The significant increase in Kim-1 expression in uninjured Sca-1^-/-^ kidneys and 7 days post IRI was confirmed by Western blot (Fig 3B). In wild-type animals, *Sca-1* expression was significantly increased by day 7 post-IRI (Fig 3C), coincident with recovery of renal function (Fig 2A) and pathological (Figs 2A and 3D) kidney injury indices. In contrast, Sca-1^-/-^ kidneys displayed increased tubule damage 7 days post injury compared to controls (Fig 3D), consistent with the rise in serum creatinine (Fig 2A). These results suggest that Sca-1 plays an important role in maintenance of epithelial cell function under baseline conditions and in the recovery phase of IRI.

### Sca-1 interacts with TβRI and TβRII in renal proximal tubular cells

**Fig 4:**
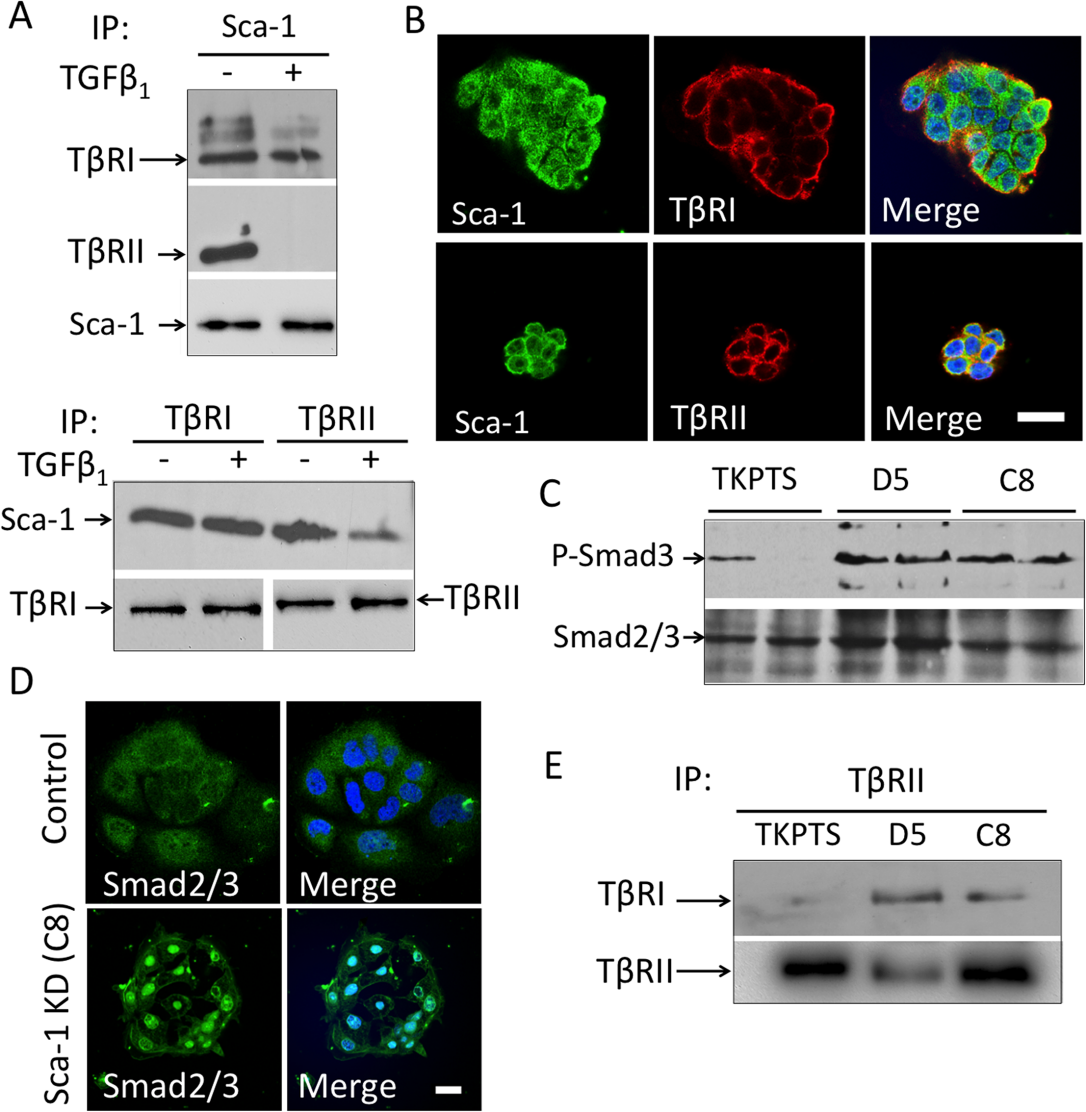
Sca-1 interacts with the TGFβ signaling pathway in mouse proximal tubule epithelial (TKPTS) cells. (A) Immunoprecipitation of TβRI and TβRII from lysates of serum-starved normal TKPTS cells by anti-Sca-1 antibody. Both TRI and TRII were immunoprecipitated with anti-Sca-1 in the absence of TGFβ_1,_ but only TβRI co-precipitated with Sca-1 in the presence of TGFβ_1_. *Lower panel*, reciprocal immunoprecipitations displayed the same trend. A representative experiment, one of three, is shown. Equal loading of samples was reflected in observed levels of Sca-1 (*upper panel*) and TβRI (*lower panel*). (B) Co-localization of Sca-1 with TβRI and TβRII in TKPTS cells. Cells were stained with antibodies against Sca-1 (green), TβRI or TβRII (red). Colocalization of Sca-1 and TR can be seen in focal surface membrane regions as well as intracellularly (yellow staining in merged images). (C) Western blots showing Smad3 phosphorylation (p-Smad3) in normal TKPTS and the Sca-1 silenced cells D5 and C8. Smad2/3 expression was used as loading control. Scale bar = 20μm. The differences in p-Smad3 in control cells perhaps reflect stochastic baseline variations in replicate confluency. Averages of p-Smad/total Smad ratios of duplicate samples from TKPTS, D5 and C8 were 0.33, 1.55 and 1.76, respectively. (D) Loss of Sca-1 expression increased Smad2/3 nuclear localization in C8 cells. Immunostaining of control TKPTS and C8 (Sca-1 KD) cells showing Smad2/3 localization (green), with actin detected with Alexa555 phalloidin (red), and nuclei labeled with DAPI (blue). Scale bar = 20μm. (E) A representative experiment, one of two, of a Western blot of TRII immunoprecipitates from serum-starved TKPTS, D5, and C8 cells, detected with anti-TRI antibody. Silencing Sca-1 in D5 and C8 cells resulted in constitutive TRI/TRII complex formation in the absence of ligand.

Previous studies showed that constitutive activation of TβRI in tubular epithelium caused AKI [28] and that deletion of TβRII in proximal tubules attenuated toxic renal injury [29]. Sca-1 has been shown to enhance tumorigenesis of a mammary adenocarcinoma cell line by binding TβRI but not TβRII [30]. We determined if Sca-1 on mouse proximal tubule (TKPTS) cells interacts with TGFβ receptors. In contrast to the previous study in mammary cells, both TβRI and TβRII co-immunoprecipitated with Sca-1 in TKPTS cells in the absence of ligand (Fig 4A; upper panel), and reciprocally, each receptor immunoprecipitated Sca-1 (Fig 4A; lower panel). TGFβ_1_ did not affect interaction of Sca-1 with TβRI, but markedly reduced interaction with TβRII (Fig 4A). Consistent with the immunoprecipitation studies, Sca-1 protein co-localized in TKPTS cells with TβRI and TβRII [31], on the cell surface or internalized, in the absence of ligand (Fig 4B).

### Canonical smad signaling is constitutive in Sca-1 silenced renal proximal tubular cells

To determine the functional consequences of Sca-1/TβR interactions, we examined downstream Smad signaling in wild type and Sca-1-silenced TKPTS cells. The latter cells were generated by viral infection of Sca-1-specific shRNA constructs, resulting in two cell lines, D5 and C8, that had more than 80% reduction in *Sca-1* mRNA (S2B Fig) and no Sca-1 protein was detected by Western blot (S2C Fig), when compared with wild type cells (S2A–S2C Fig). As in uninjured Sca-1^-/-^ kidneys, Kim-1 protein was significantly upregulated in D5 and C8 cells vs. wild type TKPTS (S3A Fig). Minimal phospho-activation of Smad3 (p-Smad3) was detected in serum-starved wild type TKPTS cells in the absence of ligand, but silencing of Sca-1 in D5 and C8 cell lines increased p-Smad3 levels by ~ 5-fold (Fig 4C). Consistently, Smad2/3 displayed a predominantly cytoplasmic localization in wild type cells but was primarily nuclear in Sca-1-silenced cells (Fig 4D). The increase in p-Smad3 and nuclear localization of Smad2/3 in Sca-1-silenced cells in the absence of ligand, suggests that TGFβ signaling is constitutive in these cells. We then tested if TβRI and TβRII form a complex in serum-starved Sca-1-silenced cells. As expected, immunoprecipitating TβRII resulted in barely detectable TβRI in wild-type TKPTS cells (Fig 4E) in the absence of ligand. In contrast, the TβRI/TβRII complex formed spontaneously in D5 and C8 cells (Fig 3E). Collectively, these data show that knockdown of Sca-1 in mouse proximal tubules results in ligand-independent formation of a signaling TβRI/TβRII complex.

### Selective regulation of TGFβ signaling by Sca-1 in proximal tubule cells

**Fig 5:**
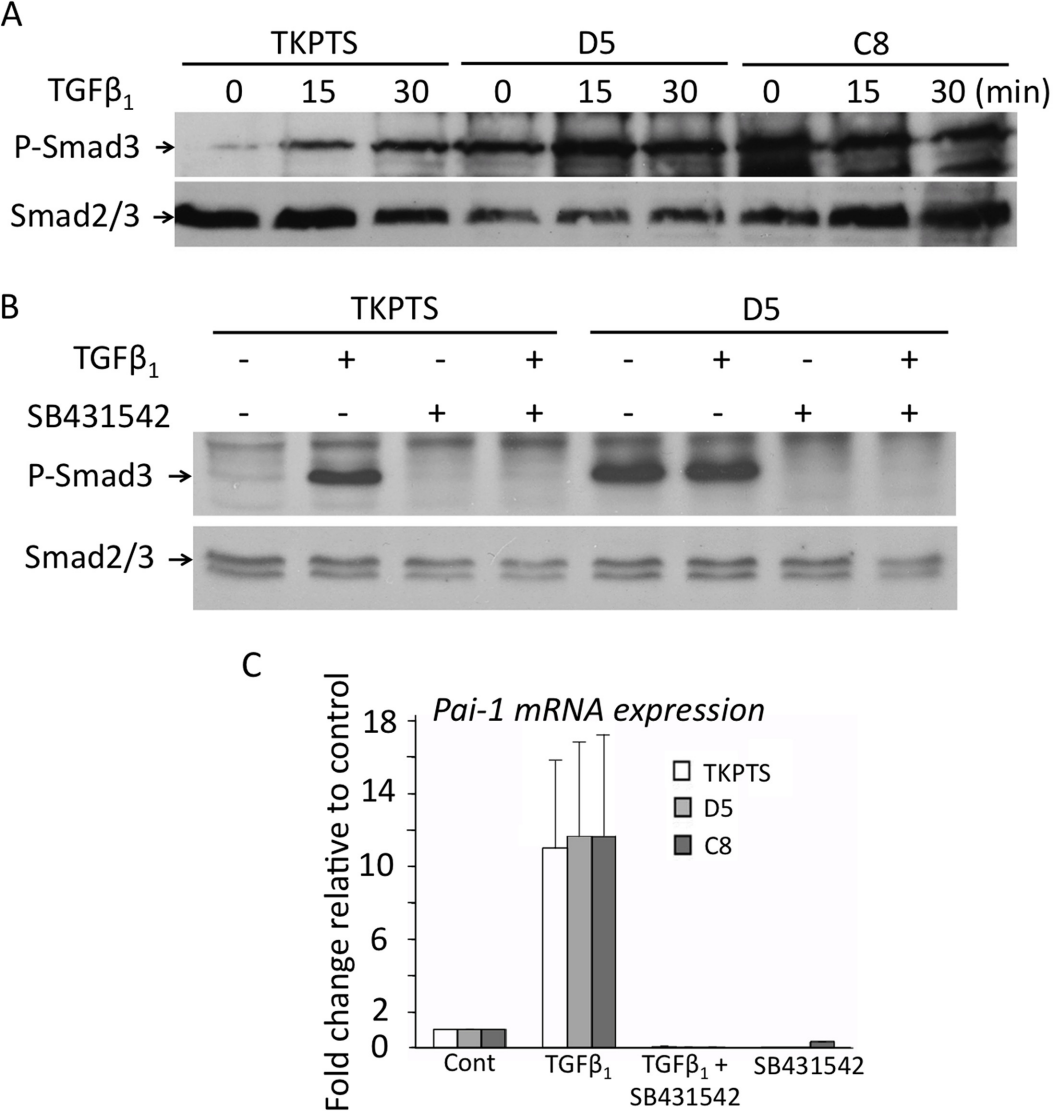
Sca-1 regulates TGFβ signaling in proximal renal tubule cells. (A) Western blots from a representative experiment, one of three conducted, showing phospho-Smad3 (p-Smad3) in untreated cell lysates from serum-starved control and Sca-1 silenced D5 and C8 cells and after exposure to TGFβ_1_ for 15 or 30 min. Smad2/3 detection served as a loading control. (B) Blocking TβRI activity abrogates ectopic Smad3 activation in Sca-1 silenced cells. TKPTS or Sca-1 silenced cells (D5) were serum starved and pretreated with SB431542 for 30 minutes prior to addition of TGFβ_1_. Cell lysates were analyzed by Western blot for detection of p-Smad3, with Smad2/3 used to control for protein loading. (C) Histograms (mean+sd, n = 3) showing *Pai-1* mRNA levels in TKPTS, D5, and C8 cells at baseline and following treatment with TGFβ_1_ and/or SB431542.

We next compared the response of wild type, D5-, and C8 TKPTS cells to the TGFβ_1_ ligand. Cells were incubated with recombinant TGFβ_1_ for various time periods, and Smad3 phosphorylation analyzed by western blotting. As noted earlier (Fig 4C), p-Smad3 was constitutively expressed in D5 and C8 cells but minimally in wild type TKPTS in the absence of ligand (Fig 5A). TKPTS cells showed a time-dependent increase in p-Smad3 in response to TGFβ_1_ (Fig 5A). However, minimal changes in p-Smad3 levels took place in D5 and C8 cells following exposure to TGFβ_1_ (Fig 5A). Preincubation of wild type TKPTS cells with the TβRI inhibitor SB431542 [32] blocked TGFβ_1_-dependent phosphorylation of Smad3 as well as ligand-independent Smad3 phosphorylation in Sca-1-silenced cells (Fig 5B), indicating that ectopic activation of Smad3 in absence of Sca-1 was also TβRI-mediated.

mRNA and protein levels of plasminogen activator inhibitor-1 (Pai-1) did not change in Sca-1 silenced D5 and C8 proximal tubular epithelial cells (Fig 5C and S3A Fig). TGFβ_1_ also induces *Pai-1* mRNA expression [33] via a TGFβ_1_-directed ERK1/2 signaling [34]. In response to TGFβ_1_, *Pai-1* mRNA rose to equivalent levels in wild type and Sca-1-silenced cells (Fig 5C), suggesting that Sca-1 does not regulate this arm of TGFβ_1_ signaling. To assess if Sca-1/TβR also regulates noncanonical TGFβ_1_-directed p38 signaling, activation of p38 MAPK was examined in Sca-1^-/-^ kidneys before and after IRI as well as in Sca-1-silenced D5 and C8 cells. We found no significant change in phosphorylated p38 MAPK in Sca-1^-/-^ kidneys or Sca-1 silenced proximal tubular epithelial cells (S3B and S3C Fig), suggesting that Sca-1 mainly influences TGFβ_1_-directed canonical Smad signaling in kidney epithelium.

### Smad signaling in kidneys of Sca-1^-/-^ mice after IRI

**Fig 6:**
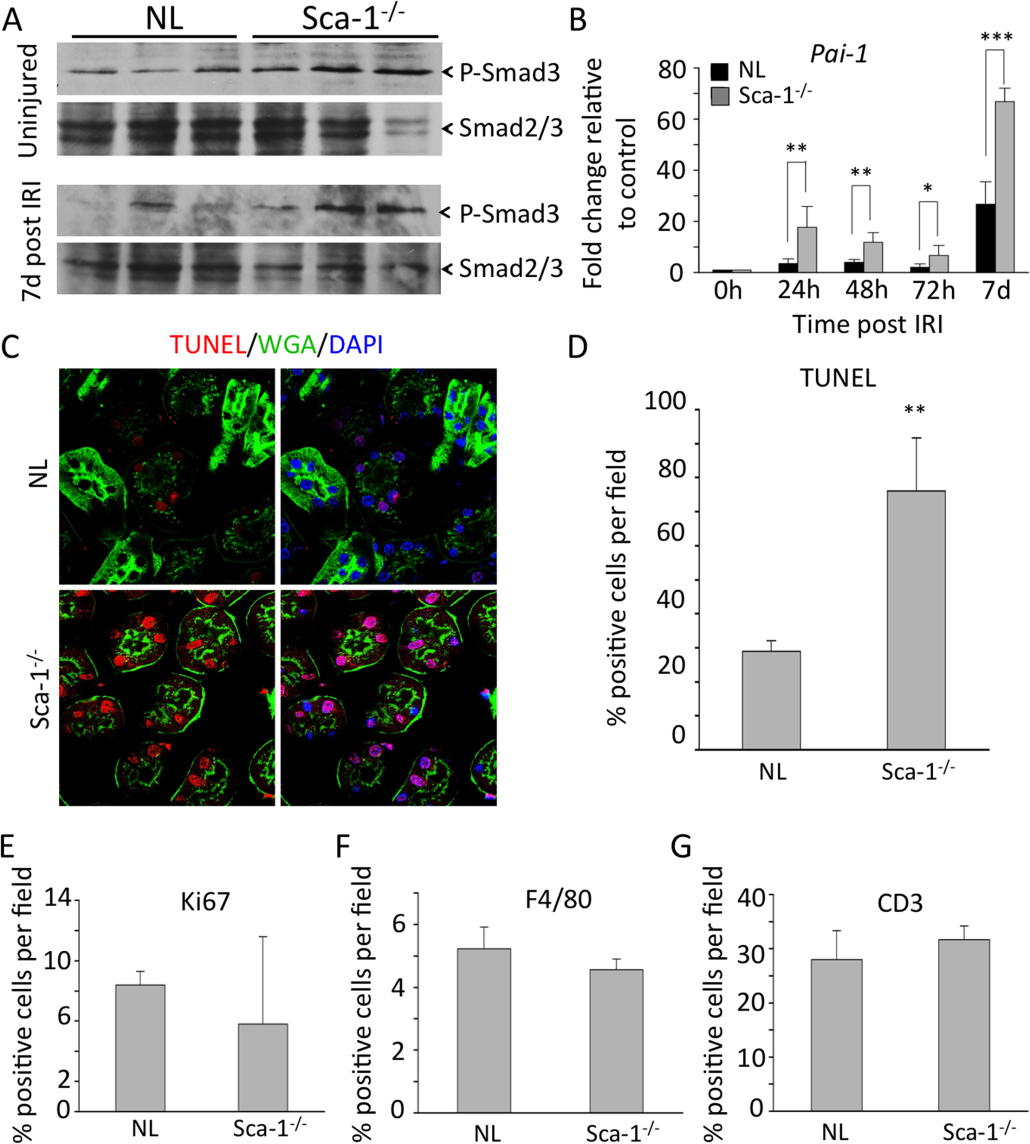
Misregulation of TGFβ pathway in Sca1^-/-^ mice after IRI. (A) Smad3 phosphorylation in normal and Sca-1^**-/-**^ kidneys prior to and 7 days after IRI (samples are from 3 animals in each case). Smad2/3 was used to control for protein loading. Quantitation of p-Smad/total Smad ratios at baseline using ImageJ revealed a 2.5-fold increase in p-smad levels in uninjured Sca-1^**-/-**^ vs. NL kidneys but the difference did not reach statistical significance (p = 0.06). However, a similar comparison at d7 post injury revealed a statistically significant increase in p-smad in Sca-1^**-/-**^ vs. NL kidneys (p = 0.0308). (B) mRNA expression of *Pai-1* in normal and Sca1^**-/-**^ kidneys after IRI. p = 0.0031 (24h), p = 0.0097 (48h), p = 0.0329 (72h), p = 0.0001 (7d). (C) Confocal imaging of TUNEL staining on sections from normal and Sca1^**-/-**^ kidneys 7 days post-IRI. Tissue was counterstained with DAPI to detect nuclei (blue) and wheat germ agglutinin (green) to differentiate between nephron epithelium and stromal cells. (D) Quantification of TUNEL- positive nephron epithelium in normal and Sca1^**-/-**^ kidneys sections shows a significant increase in TUNEL-positive cells in Sca1^**-/-**^ kidneys, p = 0.0125. (E) Quantification of Ki67 marker detection in nephron epithelium 7 days post IRI. No differences are found. (F, G) Quantification of F4/80 (F) and CD3 (G) positive cells from normal and Sca1^**-/-**^ kidney sections 7 days post IRI. Sections from 3 animals per genotype were quantified. No differences were found.

Levels of pSmad3 trended to be higher in uninjured kidneys of Sca1^-/-^ animals vs. controls at baseline (Fig 6A), but the differences did not reach statistical significance (p = 0.06). A similar trend was also observed during the first 72 hours following IRI (data not shown). However, by day 7 post-IRI, kidneys from Sca-1^-/-^ animals had significantly higher levels of phospho-Smad3 compared to wild-type kidneys (Fig 6A), as well as increased mRNA expression of *Pai-1* (Fig 6B). Comparisons between wild type and Sca-1^-/-^ animals at 7 days post-IRI, showed a significant increase in the number of apoptotic (TUNEL-positive) cells in Sca-1^-/-^ tubular epithelium (Fig 6C and 6D; p = 0.0125), but no significant change in cell proliferation (Ki67-positive tubular epithelial cells) (Fig 6E). In addition, there was no significant difference in the number of infiltrating F4/80 positive macrophages or CD3 positive T-cells in the kidneys of Sca-1^-/-^ animals 7 days post-IRI when compared to control kidneys (Fig 6F and 6G). Taken together, these data suggest that aberrant activation of canonical Smad signaling in injured Sca-1^-/-^ kidney epithelium likely accounts for increased epithelial cell apoptosis observed following IRI.

## Discussion

TGFβ signaling is a key mediator of renal scarring that ultimately leads to kidney failure [35]. Overexpression of TGFβ in the nephron can lead to cell death in proximal tubules [28] and interstitial fibrosis [36], while blocking TGFβ activity can reduce injury after AKI[37]. TGFβ1 mediates progressive renal fibrosis by inducing cell cycle arrest [38], and by stimulating the synthesis of several key fibrotic genes, such as those encoding Pai-1, collagens, fibronectin, connective tissue growth factor (CTGF), and tissue inhibitor of metalloproteinases, thus enhancing ECM production while inhibiting its degradation [39, 40]. TGFβ may also mediate renal fibrosis by inducing the transformation of tubular epithelial cells into myofibroblasts through epithelial-mesenchymal transition (EMT) [41].

In this study, we show that Sca-1 protein is heavily expressed in specific renal tubular epithelial segments, especially of the proximal tubule, a nephron segment highly sensitive to IRI. Sca-1 protein expression was examined using transgenic mice, since Sca-1 antibodies are less than ideal for Western analysis and immunostaining of whole organs due to redundancy among the proteins encoded by *Ly6* genes [11]. We find that epithelial Sca-1 plays an important homeostatic function under baseline conditions. In its absence or deficiency, kidney tubular epithelium showed elevated levels of Kim-1 and Ngal, suggesting that it is more susceptible to injury. Indeed, in a model of unilateral IRI and contralateral nephrectomy, Sca-1^-/-^ animals had elevated serum creatinine, increased expression of Kim-1 and Ngal, renal tubular epithelial cell injury and increased apoptosis. We did not find a significant increase in infiltrating macrophages or CD3^+^ lymphocytes in Sca-1^-/-^ vs. control animals, suggesting that Sca-1 null leukocytes do not play a significant role in renal injury. We traced the mechanism of kidney epithelial cell injury to upregulation of epithelial TGFβ receptor signaling. In cultured renal proximal tubular epithelial cells, Sca-1 interacted with TβRI and TβRII in the absence of ligand. In presence of ligand, Sca-1/TβRI interaction was maintained but that between Sca-1 and TβRII was lost. In Sca-1 deficiency states (in null animals or silenced tubular cells), upregulation of TGFβ receptor signaling was primarily mediated via increased canonical Smad signaling, which paralleled the rise in Kim-1 levels, suggesting a cause and effect relationship. There was no contribution detected from the noncanonical TβR-mediated MEK/ERK or p38 pathway [42].

TGFβ signaling is initiated when this ligand binds the high affinity serine/threonine kinase TβRII, allowing TβRII to complex with and activate the low affinity TβRI, which in turn promotes serine phosphorylation of Smad2/3, their association with Smad4 and translocation to the nucleus, where they trigger transcription of profibrotic genes [40]. Our data show that under baseline conditions, Sca-1 binds to both TβRII and TβRI in the absence of ligand, but only to TβRI in its presence. In contrast to our findings in renal epithelium, Sca-1 did not bind TβRII and bound only to TβRI in the absence of ligand in a mammary adenocarcinoma cell line [30], suggesting that Sca-1 interaction with TGFβ receptors is cell context-specific. Association of TβRI with TβRII is mediated by the ectodomain of each [43], a three-fingered protein domain (TFPD) also found in Sca-1 and other Ly6 protein family members [44]. A distinct site in TβRII ectodomain is used to bind ligand. Our data in renal epithelium suggest that Sca-1 may occupy the same (or an overlapping) TGFβ binding site in TβRII, and is thus displaced in presence of TGFβ. Superimposing the TFPDs of TβRII and TβRI using Chimera [17] show that the region in TβRI corresponding to the TGFβ binding-site in TβRII remains accessible to Sca-1 in the ternary TGFβ /TβRII/TβRI complex.

Functionally, Sca-1 suppressed canonical Smad signaling in uninjured or injured renal epithelium, but did not alter TGFβ-directed noncanonical signaling via ERK1/2 or p38 MAPK. These data suggest the following model: in the basal state, binding of epithelial Sca-1 to TβRI and TβRII keeps the two receptors apart on the cell surface. Following ischemic injury, induced TGFβ displaces Sca-1 from TβRII allowing its phospho-activation perhaps by Src [42]. However, continued occupancy of TβRI by Sca-1 prevents its phospho-activation by TβRII, perhaps caused by steric or allosteric effects, thus preventing TβRI-mediated canonical Smad signaling. TGFβ-bound TβRII can still activate downstream noncanonical MAPK signaling [42]. Taken together, our findings suggest that inhibition of canonical Smad signaling accounts for the role of epithelial Sca-1 in the preservation and recovery of renal function. Consistent with this interpretation is data showing that homozygous knockout of Smad3 protects against ischemic AKI in mice [45], and expression of a constitutively active form of TβRI in the proximal tubule resulted in epithelial cell injury and apoptosis [28].

Sca-1 is located within a cluster of related Ly6 genes on mouse chromosome 15, which is syntenic to human chromosome 8q24.3 [11, 46]. The segment containing Sca-1 (Ly6A) was deleted between mouse and rat speciation, thus no obvious Sca-1 homolog is known in humans. Given the important roles Sca-1 plays in mediating stem- and differentiated cell stress responses in mice, it is likely that these roles are assumed by one or a number of the eleven remaining Ly6-related genes on human chromosome 8 [46]. The identity of the functional homolog(s) of Sca-1 in humans and whether it also forms Ly6/TβR regulatory complexes remains to be determined.

## Supporting Information

S1 Fig Tubule expression of Sca-1 in adult kidney. (A-C) Cells of the Loop of Henle labeled with Tamm-Horsfall protein (THP, A) co-expressed the Sca-1-EGFP transgene (B), merged image in (C). (D-F) Distal tubules labeled with Calbindin 1 (Calb1, D) also expressed Sca-1 (E), merged image in (F). (G-I) Collecting ducts stained with rhodamine labeled *dolichos biflorus* agglutinin (DBA, G) did not display expression of the Sca-1-EGFP transgene (H), merged image in (I). Scale bar = 20μm.
(TIF)

S2 Fig shRNA knockdown of Sca-1 in TKPTS cells. (A) Sca-1 protein (green) expression on non-permeabilized TKPTS cells. Nucleus stained in blue with DAPI. (B) Histograms (mean+sd) of real-time PCR from control TKPTS cells and three Sca-1 shRNA stable knockdown cell lines. Three independent replicates were tested for Sca-1 expression from control and shRNA infected cell lines. Cell lines D5 and C8 displayed the most robust reduction of Sca-1 mRNA expression. (C) Western blot analysis of Sca-1 shRNA stable knockdown cell lines. Protein lysates from control TKPTS and C9, D5, and C8 knockdown cell lines were assessed for Sca-1 protein expression by Western blot. Cell lines D5 and C8 showed a significant reduction in Sca-1 protein. α-tubulin was used to control for protein loading.
(TIF)

S3 Fig Kim-1, Pai-1 expression and p38 activity Sca-1-deficient kidney cells. (A) Western blots showing Kim-1 levels in normal TKPTS cells, and in Sca-1 silenced D5, and C8 cells. α-Tubulin was used to control for protein loading. Kim-1 protein was clearly increased in Sca-1 silenced cells in the absence of TGFβ_1._ No changes were detected in Pai-1 protein. Similar data were obtained in two other experiments. (B) Western detection of phospho-p38 in normal and Sca1^-/-^ kidneys from three animals in each case before and 7 days post-IRI. p-p38/p38 ratios in NL and Sca-1-/- kidneys before or at 7days post injury were not different (p = 0.329 and p = 0.131, respectively). (C) Western detection of phosho-p38 in TKPTS and the Sca-1 silenced cell lines D5 and C8. p-p38/p38 ratios for wild type, D5 and C8 were 1.44, 1.74 and 1.77, respectively. One of two experiments is shown.
(TIF)

## References

[1] Susantitaphong P, Cruz DN, Cerda J, Abulfaraj M, Alqahtani F, Koulouridis I., et al (2013) World incidence of AKI: a meta-analysis. Clin J Am Soc Nephrol 8: 1482–1493. 10.2215/CJN.00710113 23744003 PMC3805065

[2] Ali T, Khan I, Simpson W, Prescott G, Townend J, Smith W., et al (2007) Incidence and outcomes in acute kidney injury: a comprehensive population-based study. J Am Soc Nephrol 18: 1292–1298. 17314324 10.1681/ASN.2006070756

[3] Renal Data System: USRDS (2009) Annual Data Report: Atlas of End-Stage Renal Disease in the United States. National Institutes of Health, National Institute of Diabetes and Digestive and Kidney Diseases Bethesda, MD.

[4] Houchens RL, Elixhauser A (2006) Using the HCUP Nationwide Inpatient Sample to Estimate Trends (updated for 1988–2004). HCUP Methods Series Report #2006–05 (online). US Agency for Healthcare Research and Quality. Available at http://www.hcup-us.ahrq.gov/reports/methods.jsp.

[5] Chawla LS, Amdur RL, Amodeo S, Kimmel PL, Palant CE (2011) The severity of acute kidney injury predicts progression to chronic kidney disease. Kidney Int 79: 1361–1369. 10.1038/ki.2011.42 21430640 PMC3257034

[6] Coca SG, Yusuf B, Shlipak MG, Garg AX, Parikh CR (2009) Long-term risk of mortality and other adverse outcomes after acute kidney injury: a systematic review and meta-analysis. Am J Kidney Dis 53: 961–973. 10.1053/j.ajkd.2008.11.034 19346042 PMC2726041

[7] Venkatachalam MA, Griffin KA, Lan R, Geng H, Saikumar P, Bidani A. K. (2010) Acute kidney injury: a springboard for progression in chronic kidney disease. Am J Physiol Renal Physiol 298: F1078–1094. 10.1152/ajprenal.00017.2010 20200097 PMC2867413

[8] Ichimura T, Hung CC, Yang SA, Stevens JL, Bonventre JV (2004) Kidney injury molecule-1: a tissue and urinary biomarker for nephrotoxicant-induced renal injury. Am J Physiol Renal Physiol 286: F552–563. 14600030 10.1152/ajprenal.00285.2002

[9] Yang L, Besschetnova TY, Brooks CR, Shah JV, Bonventre JV (2010) Epithelial cell cycle arrest in G2/M mediates kidney fibrosis after injury. Nat Med 16: 535–543, 531p following 143. 10.1038/nm.2144 20436483 PMC3928013

[10] Nath KA, Croatt AJ, Haggard JJ, Grande JP (2000) Renal response to repetitive exposure to heme proteins: chronic injury induced by an acute insult. Kidney Int 57: 2423–2433. 10844611 10.1046/j.1523-1755.2000.00101.x

[11] Holmes C, Stanford WL (2007) Concise review: stem cell antigen-1: expression, function, and enigma. Stem Cells 25: 1339–1347. 17379763 10.1634/stemcells.2006-0644

[12] Ito CY, Li CY, Bernstein A, Dick JE, Stanford WL (2003) Hematopoietic stem cell and progenitor defects in Sca-1/Ly-6A-null mice. Blood 101: 517–523. 12393491 10.1182/blood-2002-06-1918

[13] Welm BE, Tepera SB, Venezia T, Graubert TA, Rosen JM, Goodell M. A. (2002) Sca-1(pos) cells in the mouse mammary gland represent an enriched progenitor cell population. Dev Biol 245: 42–56. 11969254 10.1006/dbio.2002.0625

[14] Mitchell PO, Mills T, O'Connor RS, Kline ER, Graubert T, Dzierzak E., et al (2005) Sca-1 negatively regulates proliferation and differentiation of muscle cells. Dev Biol 283: 240–252. 15901485 10.1016/j.ydbio.2005.04.016

[15] Epting CL, Lopez JE, Pedersen A, Brown C, Spitz P, Ursell P. C., et al (2008) Stem cell antigen-1 regulates the tempo of muscle repair through effects on proliferation of alpha7 integrin-expressing myoblasts. Exp Cell Res 314: 1125–1135. 18073129 10.1016/j.yexcr.2007.11.010 PMC2292416

[16] McQualter JL, Brouard N, Williams B, Baird BN, Sims-Lucas S, Yuen K., et al (2009) Endogenous fibroblastic progenitor cells in the adult mouse lung are highly enriched in the sca-1 positive cell fraction. Stem Cells 27: 623–633. 10.1634/stemcells.2008-0866 19074419

[17] Bonyadi M, Waldman SD, Liu D, Aubin JE, Grynpas MD, Stanford W. L. (2003) Mesenchymal progenitor self-renewal deficiency leads to age-dependent osteoporosis in Sca-1/Ly-6A null mice. Proc Natl Acad Sci U S A 100: 5840–5845. 12732718 10.1073/pnas.1036475100 PMC156288

[18] Bailey B, Fransioli J, Gude NA, Alvarez R Jr., Zhang X, Gustafsson A. B., et al (2012) Sca-1 knockout impairs myocardial and cardiac progenitor cell function. Circ Res 111: 750–760. 10.1161/CIRCRESAHA.112.274662 22800687 PMC3463406

[19] Kafadar KA, Yi L, Ahmad Y, So L, Rossi F, Pavlath G. K. (2009) Sca-1 expression is required for efficient remodeling of the extracellular matrix during skeletal muscle regeneration. Dev Biol 326: 47–59. 10.1016/j.ydbio.2008.10.036 19059231 PMC2659587

[20] Zhou H, Bian ZY, Zong J, Deng W, Yan L, Shen D. F., et al (2012) Stem cell antigen 1 protects against cardiac hypertrophy and fibrosis after pressure overload. Hypertension 60: 802–809. 10.1161/HYPERTENSIONAHA.112.198895 22851736

[21] van de Rijn M, Heimfeld S, Spangrude GJ, Weissman IL (1989) Mouse hematopoietic stem-cell antigen Sca-1 is a member of the Ly-6 antigen family. Proc Natl Acad Sci U S A 86: 4634–4638. 2660142 10.1073/pnas.86.12.4634 PMC287325

[22] Blake PG, Madrenas J, Halloran PF (1993) Ly-6 in kidney is widely expressed on tubular epithelium and vascular endothelium and is up-regulated by interferon gamma. J Am Soc Nephrol 4: 1140–1150. 8305641 10.1681/ASN.V451140

[23] Stanford WL, Haque S, Alexander R, Liu X, Latour AM, Snodgrass H. R., et al (1997) Altered proliferative response by T lymphocytes of Ly-6A (Sca-1) null mice. J Exp Med 186: 705–717. 9271586 10.1084/jem.186.5.705 PMC2199024

[24] Skrypnyk NI, Harris RC, de Caestecker MP (2013) Ischemia-reperfusion model of acute kidney injury and post injury fibrosis in mice. J Vis Exp. 10.3791/50495 PMC3854859 23963468

[25] Winslow V, Vaivoda R, Vasilyev A, Dombkowski D, Douaidy K, Stark C., et al (2014) Altered leukotriene B4 metabolism in CYP4F18-deficient mice does not impact inflammation following renal ischemia. Biochim Biophys Acta 1841: 868–879. 10.1016/j.bbalip.2014.03.002 24632148 PMC4013684

[26] Camarata T, Bimber B, Kulisz A, Chew TL, Yeung J, Simon H. G. (2006) LMP4 regulates Tbx5 protein subcellular localization and activity. J Cell Biol 174: 339–348. 16880269 10.1083/jcb.200511109 PMC2064230

[27] Schneider CA, Rasband W. S. and Eliceiri K. W. (2012) NIH Image to ImageJ: 25 years of image analysis. Nature methods 9: 671–675. 22930834 10.1038/nmeth.2089 PMC5554542

[28] Gentle ME, Shi S, Daehn I, Zhang T, Qi H, Yu L., et al (2013) Epithelial cell TGFbeta signaling induces acute tubular injury and interstitial inflammation. J Am Soc Nephrol 24: 787–799. 10.1681/ASN.2012101024 23539761 PMC3636798

[29] Gewin L, Vadivelu S, Neelisetty S, Srichai MB, Paueksakon P, Pozzi A., et al (2012) Deleting the TGF-beta receptor attenuates acute proximal tubule injury. J Am Soc Nephrol 23: 2001–2011. 10.1681/ASN.2012020139 23160515 PMC3507360

[30] Upadhyay G, Yin Y, Yuan H, Li X, Derynck R, Glazer R. I. (2011) Stem cell antigen-1 enhances tumorigenicity by disruption of growth differentiation factor-10 (GDF10)-dependent TGF-beta signaling. Proc Natl Acad Sci U S A 108: 7820–7825. 10.1073/pnas.1103441108 21518866 PMC3093514

[31] Chen YG (2009) Endocytic regulation of TGF-beta signaling. Cell Res 19: 58–70. 10.1038/cr.2008.315 19050695

[32] Inman GJ, Nicolas FJ, Callahan JF, Harling JD, Gaster LM, Reith A. D., et al (2002) SB-431542 is a potent and specific inhibitor of transforming growth factor-beta superfamily type I activin receptor-like kinase (ALK) receptors ALK4, ALK5, and ALK7. Mol Pharmacol 62: 65–74. 12065756 10.1124/mol.62.1.65

[33] Kutz SM, Hordines J, McKeown-Longo PJ, Higgins PJ (2001) TGF-beta1-induced PAI-1 gene expression requires MEK activity and cell-to-substrate adhesion. J Cell Sci 114: 3905–3914. 11719557 10.1242/jcs.114.21.3905

[34] Samarakoon R, Higgins PJ (2008) Integration of non-SMAD and SMAD signaling in TGF-beta1-induced plasminogen activator inhibitor type-1 gene expression in vascular smooth muscle cells. Thromb Haemost 100: 976–983. 19132220 PMC2963177

[35] Liu Y (2006) Renal fibrosis: new insights into the pathogenesis and therapeutics. Kidney Int 69: 213–217. 16408108 10.1038/sj.ki.5000054

[36] Koesters R, Kaissling B, Lehir M, Picard N, Theilig F, Gebhardt R., et al (2010) Tubular overexpression of transforming growth factor-beta1 induces autophagy and fibrosis but not mesenchymal transition of renal epithelial cells. Am J Pathol 177: 632–643. 10.2353/ajpath.2010.091012 20616344 PMC2913362

[37] Geng H, Lan R, Wang G, Siddiqi AR, Naski MC, Brooks A. I., et al (2009) Inhibition of autoregulated TGFbeta signaling simultaneously enhances proliferation and differentiation of kidney epithelium and promotes repair following renal ischemia. Am J Pathol 174: 1291–1308. 10.2353/ajpath.2009.080295 19342372 PMC2671361

[38] Wu CF, Chiang WC, Lai CF, Chang FC, Chen YT, Chou Y. H., et al (2013) Transforming growth factor beta-1 stimulates profibrotic epithelial signaling to activate pericyte-myofibroblast transition in obstructive kidney fibrosis. Am J Pathol 182: 118–131. 10.1016/j.ajpath.2012.09.009 23142380 PMC3538028

[39] Lan HY, Chung AC (2012) TGF-beta/Smad signaling in kidney disease. Semin Nephrol 32: 236–243. 10.1016/j.semnephrol.2012.04.002 22835454

[40] Massague J (2012) TGFbeta signalling in context. Nat Rev Mol Cell Biol 13: 616–630. 10.1038/nrm3434 22992590 PMC4027049

[41] Fragiadaki M, Mason RM (2011) Epithelial-mesenchymal transition in renal fibrosis—evidence for and against. Int J Exp Pathol 92: 143–150. 10.1111/j.1365-2613.2011.00775.x 21554437 PMC3101487

[42] Galliher AJ, Schiemann WP (2007) Src phosphorylates Tyr284 in TGF-beta type II receptor and regulates TGF-beta stimulation of p38 MAPK during breast cancer cell proliferation and invasion. Cancer Res 67: 3752–3758. 17440088 10.1158/0008-5472.CAN-06-3851

[43] Groppe J, Hinck CS, Samavarchi-Tehrani P, Zubieta C, Schuermann JP, Taylor A. B., et al (2008) Cooperative assembly of TGF-beta superfamily signaling complexes is mediated by two disparate mechanisms and distinct modes of receptor binding. Mol Cell 29: 157–168. 10.1016/j.molcel.2007.11.039 18243111

[44] Galat A, Gross G, Drevet P, Sato A, Menez A (2008) Conserved structural determinants in three-fingered protein domains. FEBS J 275: 3207–3225. 10.1111/j.1742-4658.2008.06473.x 18485004

[45] Nath KA, Croatt AJ, Warner GM, Grande JP (2011) Genetic deficiency of Smad3 protects against murine ischemic acute kidney injury. Am J Physiol Renal Physiol 301: F436–442. 10.1152/ajprenal.00162.2011 21525133 PMC3154585

[46] Lee PY, Wang JX, Parisini E, Dascher CC, Nigrovic PA (2103) Ly6 family proteins in neutrophil biology. J Leukoc Biol. 94:585–94. 10.1189/jlb.0113014 23543767

