# Supplementary figures and images for: Negative Regulation of TGFβ Signaling by Stem Cell Antigen-1 Protects against Ischemic Acute Kidney Injury
Source: PLoS One. 2015 Jun 8;10(6):e0129561. doi: 10.1371/journal.pone.0129561 (PMC4460127; doi:10.1371/journal.pone.0129561)

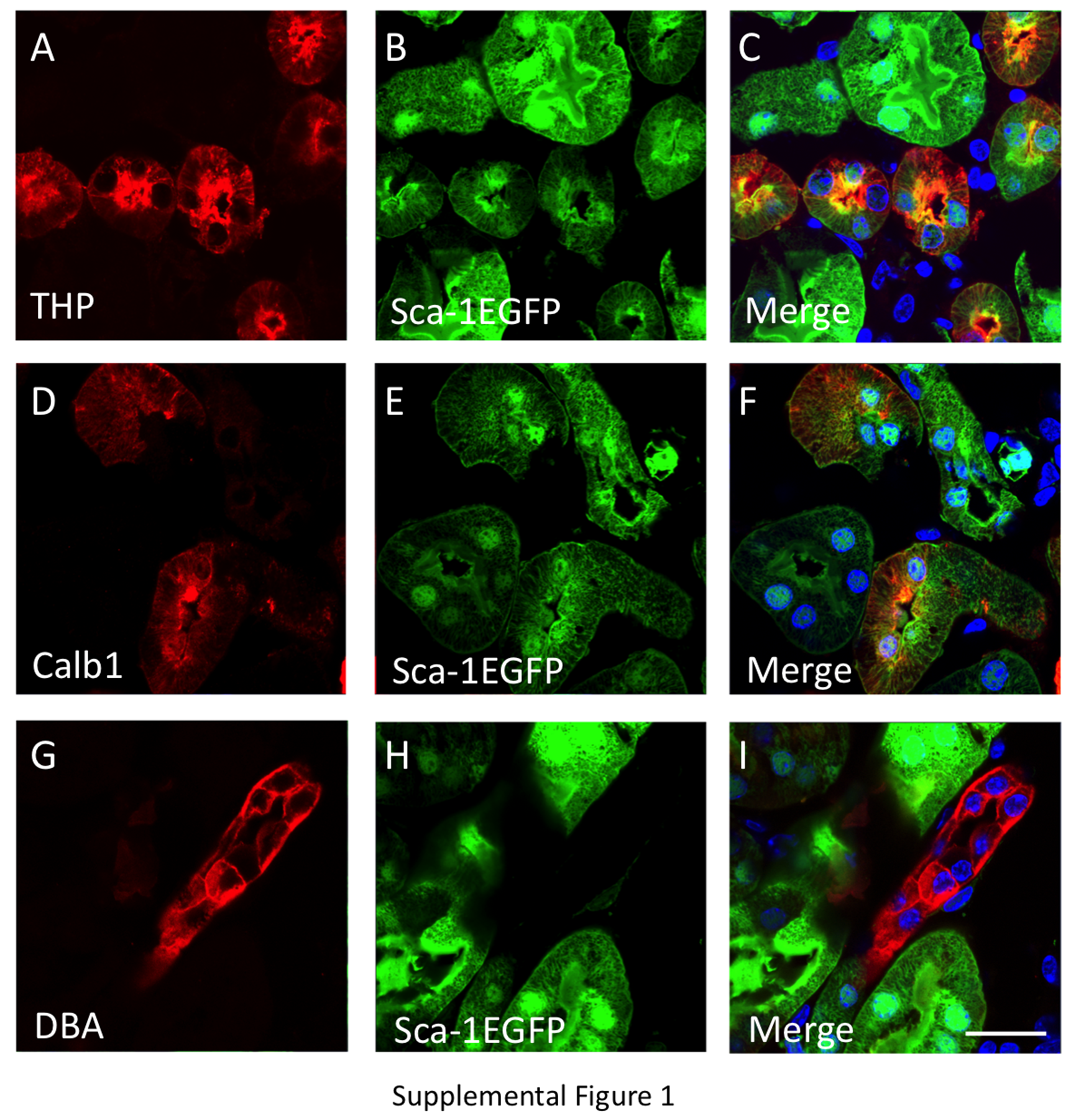

Supplement: S1 Fig — (A-C) Cells of the Loop of Henle labeled with Tamm-Horsfall protein (THP, A) co-expressed the Sca-1-EGFP transgene (B), merged image in (C). (D-F) Distal tubules labeled with Calbindin 1 (Calb1, D) also expressed Sca-1 (E), merged image in (F). (G-I) Collecting ducts stained with rhodamine labeled dolichos biflorus agglutinin (DBA, G) did not display expression of the Sca-1-EGFP transgene (H), merged image in (I). Scale bar = 20μm. (TIF) [file pone.0129561.s001.tif]

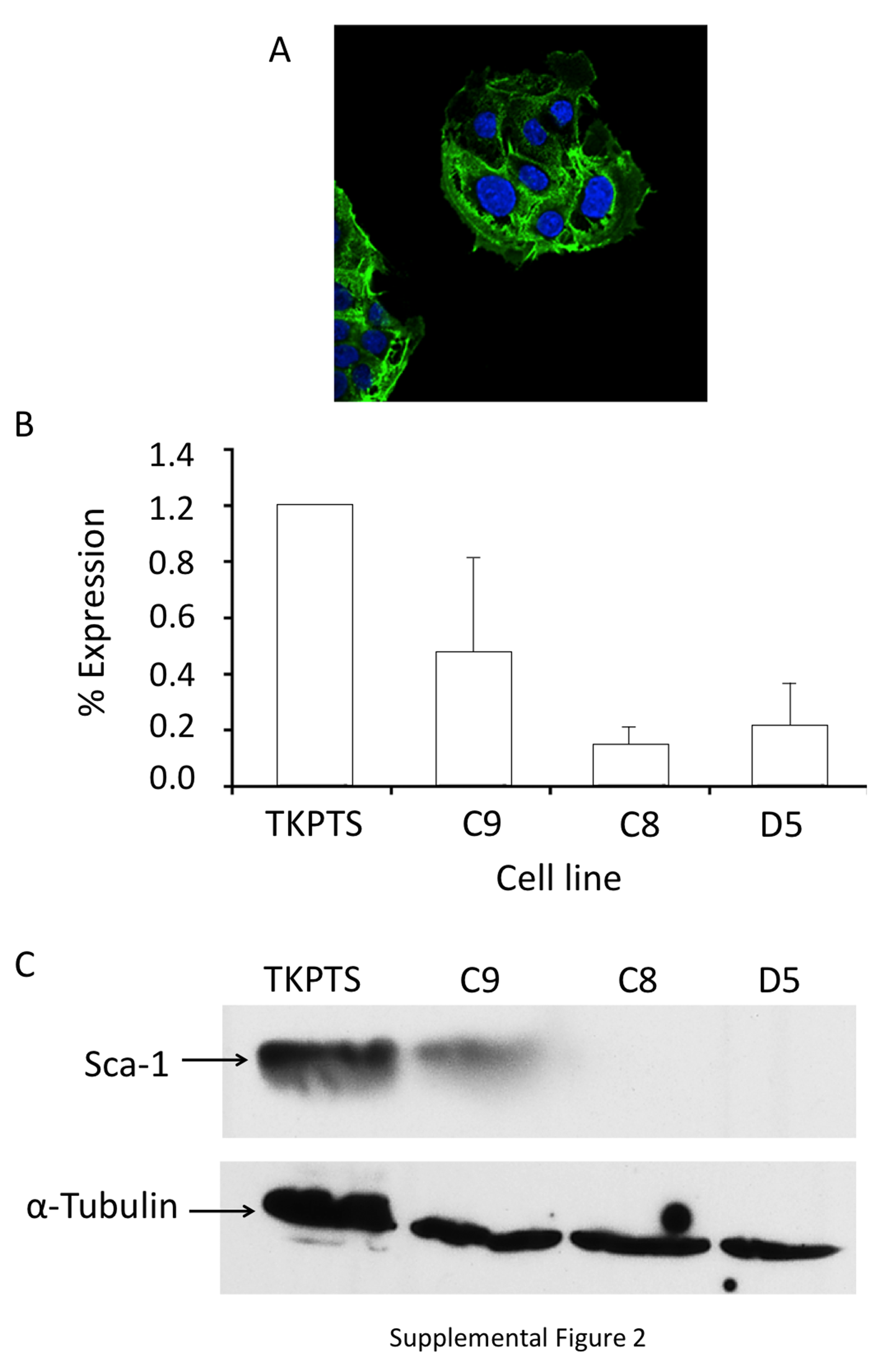

Supplement: S2 Fig — (A) Sca-1 protein (green) expression on non-permeabilized TKPTS cells. Nucleus stained in blue with DAPI. (B) Histograms (mean+sd) of real-time PCR from control TKPTS cells and three Sca-1 shRNA stable knockdown cell lines. Three independent replicates were tested for Sca-1 expression from control and shRNA infected cell lines. Cell lines D5 and C8 displayed the most robust reduction of Sca-1 mRNA expression. (C) Western blot analysis of Sca-1 shRNA stable knockdown cell lines. Protein lysates from control TKPTS and C9, D5, and C8 knockdown cell lines were assessed for Sca-1 protein expression by Western blot. Cell lines D5 and C8 showed a significant reduction in Sca-1 protein. α-tubulin was used to control for protein loading. (TIF) [file pone.0129561.s002.tif]

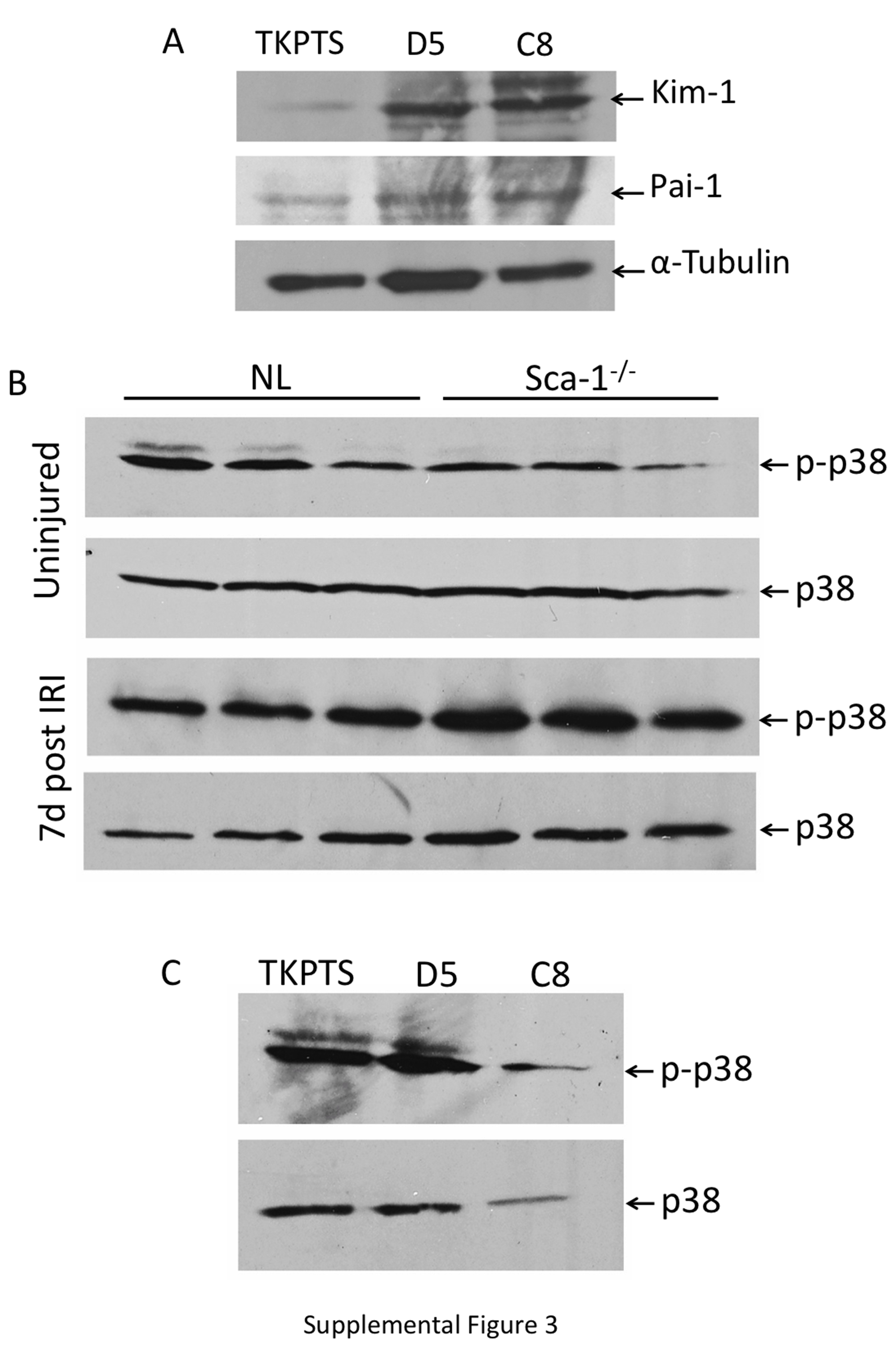

Supplement: S3 Fig — (A) Western blots showing Kim-1 levels in normal TKPTS cells, and in Sca-1 silenced D5, and C8 cells. α-Tubulin was used to control for protein loading. Kim-1 protein was clearly increased in Sca-1 silenced cells in the absence of TGFβ1. No changes were detected in Pai-1 protein. Similar data were obtained in two other experiments. (B) Western detection of phospho-p38 in normal and Sca1-/- kidneys from three animals in each case before and 7 days post-IRI. p-p38/p38 ratios in NL and Sca-1-/- kidneys before or at 7days post injury were not different (p = 0.329 and p = 0.131, respectively). (C) Western detection of phosho-p38 in TKPTS and the Sca-1 silenced cell lines D5 and C8. p-p38/p38 ratios for wild type, D5 and C8 were 1.44, 1.74 and 1.77, respectively. One of two experiments is shown. (TIF) [file pone.0129561.s003.tif]
